# Supplementary material for: Polymeric Interlayer in CdS-Free Electron-Selective Contact for Sb2Se3 Thin-Film Solar Cells
Source: Int J Mol Sci. 2023 Feb 4;24(4):3088. doi: 10.3390/ijms24043088 (PMC9964030; doi:10.3390/ijms24043088)
Supplement: Supplementary file 1 [file ijms-24-03088-s001.zip › ijms-2026188-supplementary.pdf]

Supplementary Materials:

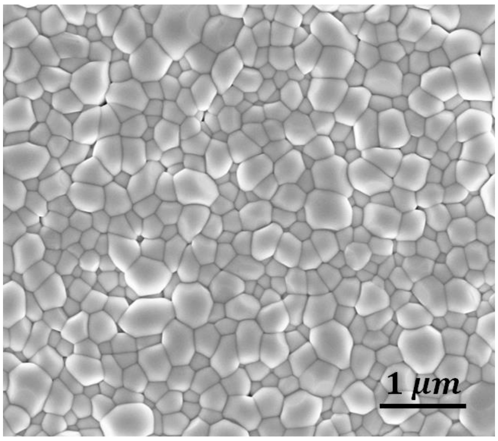

(a)

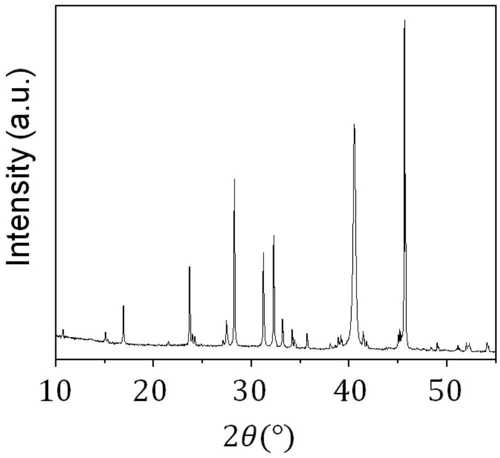

(b)

Figure S1. (**a**) SEM micrography of Sb_2Se_3 absorber layer with granular shape and pinhole presence. (**b**) XRD diffraction pattern of the same sample.

Table S1. XRF spectroscopy results of Sb_2Se_3 absorber layer.

Element	Mean Value	Standard Dev.	C.O.C (%)
Sb_2Se_3	659.9 nm	4.45 nm	0.68
Sb	34.4%	0.297%	0.86
Se	65.6%	0.297%	0.45
Mo	777.0 nm	2.55 nm	0.33

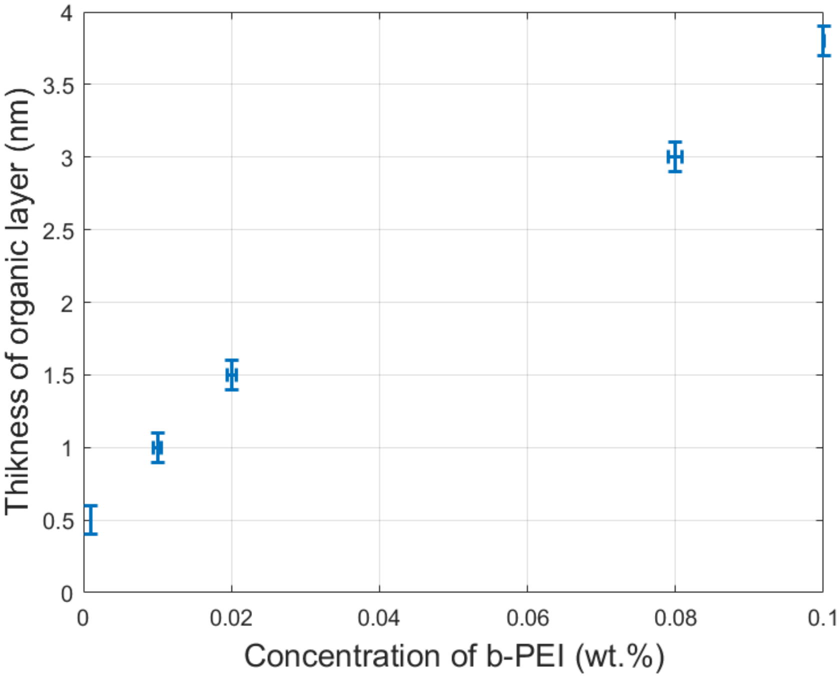

Figure S2. Linear relation between b-PEI solution concentration and polymeric interlayer thickness. Error bars for both parameters are included.

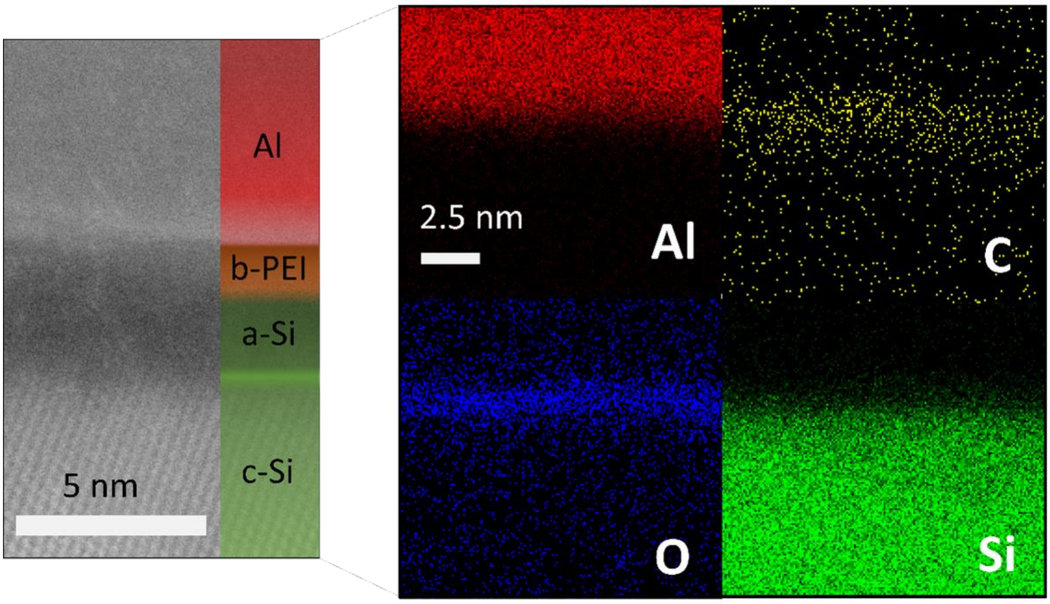

Figure S3. EDS signals of silicon (green), carbon (yellow), oxygen (blue), and aluminum (red), with their depth profile in the c-Si/b-PEI (0.01 wt.%)/Al junction.

Table S2. c-Si/b-PEI/Al contact resistivity for different b-PEI solvents.

Solvent	Molecular Dipole Moment (D) [44]	Resistivity ($\Omega \cdot cm^2$)
Toluene	0.375	$8.82 \cdot 10^{-2}$
Ethanol	1.44–1.68	$5.23 \cdot 10^{-2}$
Ethanol/water mix	1.65–1.77	$1.77 \cdot 10^{-2}$
Methanol	1.70	$0.98 \cdot 10^{-2}$

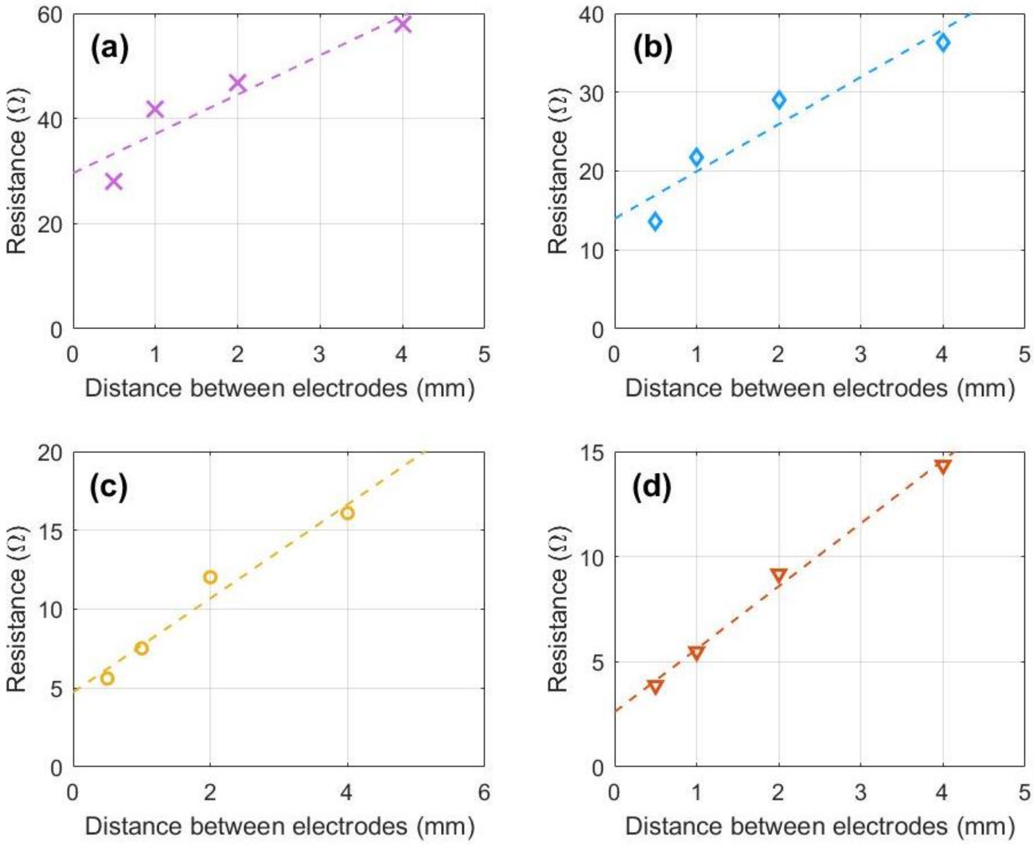

Figure S4. TLM graphs for 1nm b-PEI interlayer obtained by spin coating in (**a**) toluene, (**b**) ethanol, (**c**) ethanol/water mix, and (**d**) methanol solutions.

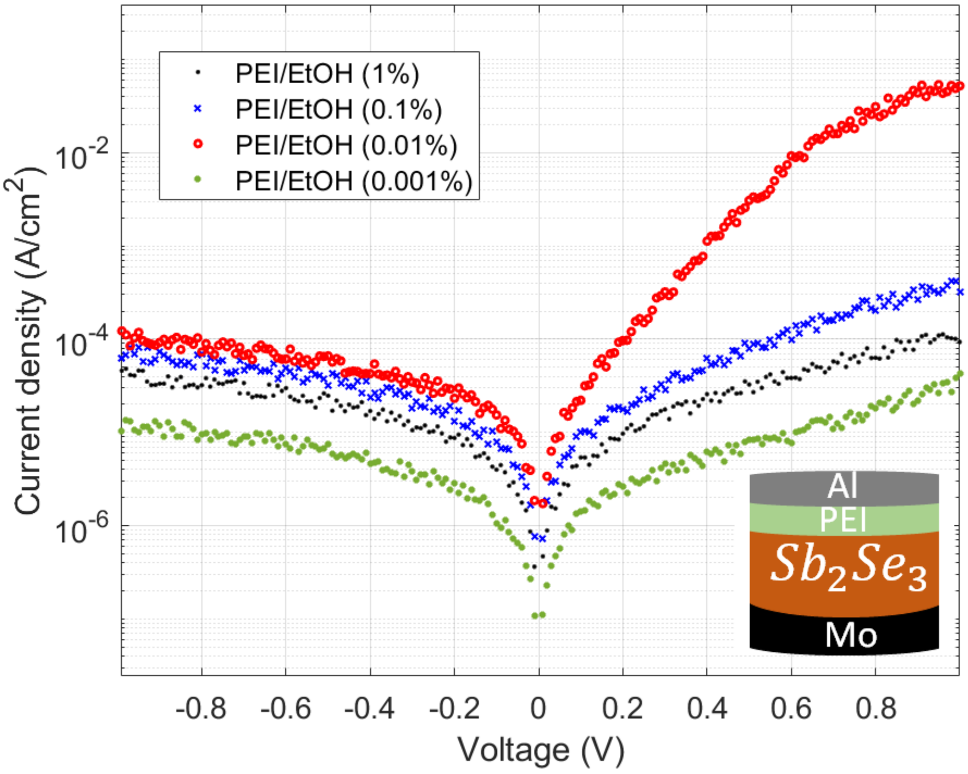

Figure S5. J-V curves of different concentrations of b-PEI as ETL in Sb_2Se_3 diodes without buffer layers.

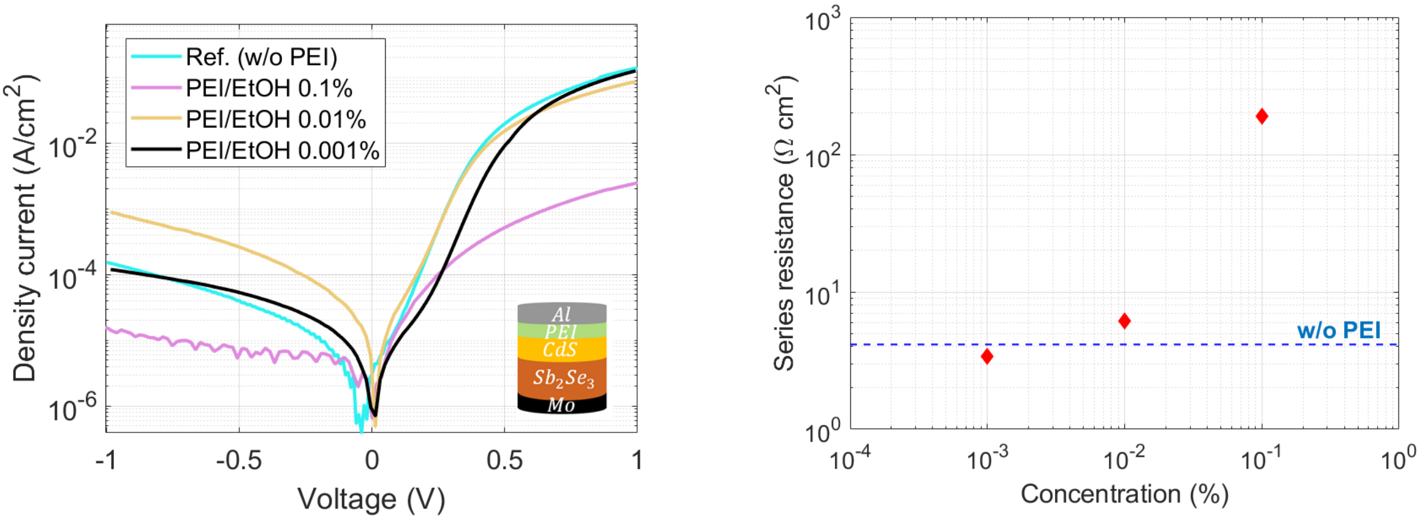

(**a**) (**b**)

Figure S6. (**a**) J-V curves of different concentrations of b-PEI as ETL in Sb_2Se_3 diodes with CdS buffer layer. (**b**) Fitted values (red diamonds) of series resistance for the J-V curves in (**a**).
